# Surface Plasmon Resonance for Cell-Based Clinical Diagnosis

**DOI:** 10.3390/s140304948

**Published:** 2014-03-11

**Authors:** Yuhki Yanase, Takaaki Hiragun, Kaori Ishii, Tomoko Kawaguchi, Tetsuji Yanase, Mikio Kawai, Kenji Sakamoto, Michihiro Hide

**Affiliations:** 1 Department of Dermatology, Division of Molecular Medical Science, Graduate School of Biomedical Science, Hiroshima University, 1-2-3, Kasumi, Minami-ku, Hiroshima 734-8551, Japan; E-Mails: yyanase@hiroshima-u.ac.jp (Y.Y.); hiragunt@hiroshima-u.ac.jp (T.H.); ishiik@hiroshima-u.ac.jp (K.I.); tomokok@hiroshima-u.ac.jp (T.K.); yanaset@hiroshima-u.ac.jp (T.Y.); mkawai02@hiroshima-u.ac.jp (M.K.); 2 Center for Microelectronic systems, Kyushu Institute of Technology, 680-4, Kawazu, Iizuka, Fukuoka, 820-8502, Japan; E-Mail: sakamoto@cms.kyutech.ac.jp

**Keywords:** surface plasmon resonance (SPR), SPR imaging (SPRI), biosensor, clinical diagnosis, diagnosis of allergy, diagnosis of cancer, optic fiber SPR

## Abstract

Non-invasive real-time observations and the evaluation of living cell conditions and functions are increasingly demanded in life sciences. Surface plasmon resonance (SPR) sensors detect the refractive index (RI) changes on the surface of sensor chips in label-free and on a real-time basis. Using SPR sensors, we and other groups have developed techniques to evaluate living cells' reactions in response to stimuli without any labeling in a real-time manner. The SPR imaging (SPRI) system for living cells may visualize single cell reactions and has the potential to expand application of SPR cell sensing for clinical diagnosis, such as multi-array cell diagnostic systems and detection of malignant cells among normal cells in combination with rapid cell isolation techniques.

## 1. Biosensors for Living Cells

The cell is the minimum unit for living creatures, whether they be bacteria or vertebrates. Non-invasive real-time observations and the evaluation of living cell conditions and functions are increasingly desired, not only for basic research in life sciences, but also for various medical practices. To date, various living cell reaction-based biosensors, such as impedance sensors [1,2], quartz crystal microbalance (QCM) sensors [3,4] and field effect transistor (FET) sensors [5,6] have been reported. Optical biosensors, surface plasmon resonance (SPR) sensors and resonant waveguide grating (RWG) sensors [7,8] also have been applied for the detection of living cell reactions in response to stimuli without any labeling. Due to the high sensitivity and the potential application for single cell imaging and endoscopic instruments, SPR sensors represent one of the potentially useful tools for basic research and clinical diagnosis. In this review, we focus on cell analysis by means of SPR and provide information on SPR sensors applied for living cells analysis and clinical diagnosis. These sensors are summarized in Table 1.

## 2. SPR Sensors

**Figure 1.**
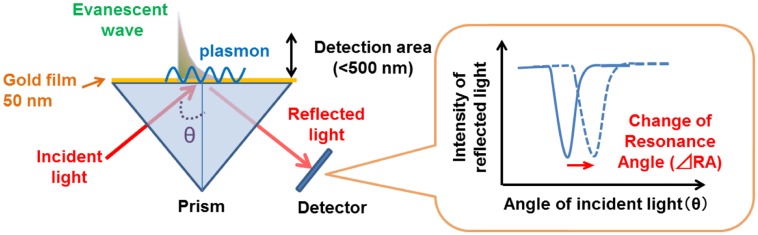
Principle of SPR sensor. Surface plasmon resonance (SPR) sensors detect a refractive index (RI) changes within a detection area (<500 nm) as a change of resonance angle (RA).

**Figure 2.**
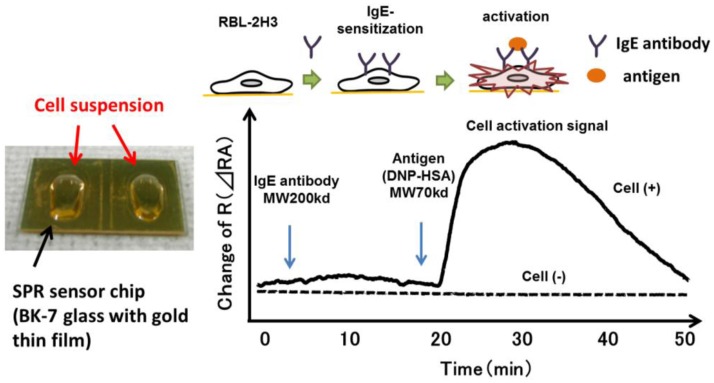
SPR signals (change of RA) obtained by binding of anti-DNP IgE and DNP-HSA to RBL-2H3 cells, which express the high affinity IgE receptor (FcεRI) on cell surface. Cells were cultured on the surface of the SPR sensor and incubated first with anti-DNP IgE and then with DNP-HSA.

SPR reflects the reflective index (RI) in the evanescent field on a metal. The resonance angle (RA) for SPR changes proportionally to the density of biological molecules in the evanescent field (<500 nm) on the other surface of the metal, whose thickness is smaller than the wavelength of the incident light. Thus, SPR can detect in a real-time manner the association and dissociation of biological molecules on a surface gold film without any labeling [9–12]. During the past two decades, SPR based-biosensors have been widely employed for label-free, real-time analyses of different biological molecules, such as antibody and antigen, receptor and ligand, and complementary DNA fragments in physiological conditions (Figure 1). In 2002, we first reported that living RBL-2H3 cells, a cognate rat mast cell line, caused an unexpectedly large increase of RA in response to biological stimuli beyond that due to a simple binding of IgE antibody to the cells (Figure 2) [13]. Large changes of RA due to cell activations have been found in other cells, such as basophils and lymphocytes obtained from human blood, and epidermal cells [14]. To employ SPR as a real-time, label-free biosensor to study cell activities in a wide range of bioscience and clinical medicine scenarios, we studied the relation of SPR signals to intracellular signal transductions, and have developed a glass-fiber SPR that detects cell reactions on the fiber tip, and a 2-dimensional SPR system that visualizes single cell reactions.

## 3. Cell Reactions/Molecules Detection by SPR

Since SPR sensors only detect changes of RI in the evanescent field on the gold surface, the objects detected by an SPR sensor should therefore be molecules in and around plasma membrane of the cells on a sensor chip. Thus, an increase in cell attachment should increase RA. Moreover, certain types of cells, including RBL-2H3 cells, and keratinocytes, show an increase in the attachment area in response to exogenous stimuli. However, the actual changes of RA by the activation of these cells were much larger than the increase in the cell attachment area. Furthermore, SPR signals (changes of RA) are tri-phasic, whereas the area of cell attachment of PAM-212 cells, a mouse keratinocyte line, simply increases during the measurement [14]. On the other hand, the inhibition of RBL-2H3 cell mobility by an act in polymerization inhibitor, cytochalasin D, partially inhibited the SPR signal in response to antigen, whereas all movement and morphological changes of the cells observed using a fluorescence microscope were stopped. Chen *et al.* demonstrated that GPCR-mediated SPR responses in CHO cells were induced by density changes on the sensor chip surface [15]. Finally, the inhibition of receptor activities by molecular engineering totally abolished SPR signals, preserving the binding activities of ligands [14,16,17]. These observations demonstrate that RI near the plasma membrane, which might reflect accumulation and rearrangement of proteins activated by intracellular signal transduction, dramatically changes in response to exogenous stimuli. As described above, the detection depth of SPR sensors is less than 500 nm from the surface of gold, much smaller than the cell height. Therefore, SPR sensors can detect RI changes near the plasma membrane with a high degree of sensitivity, whereas SPR sensors cannot detect whole cells RI changes, especially in the upper area. Recently, long-range SPR (LRSPR) sensors for living cells have been reported [18,19]. Since LRSPR sensors can enhance the detection depth to 1,000 nm or more, LRSPR sensors have the capacity for a deeper level of detection inside living cells as compared to conventional Kretschmann SPR sensors. Therefore, the analysis of RI changes in living cells using LRSPR sensors enable us to clarify the detailed mechanism of RI changes in living cells. Since the change of RI in the detection area of SPR sensors is estimated to be around 0.0004, the sensitivity of SPR sensor is enough to detect living cell reactions, such as RBL-2H3 cell sand basophils responses to antigen. However, further improvement of the sensitivity is expected to detect much smaller reactions of living cells. Several methods to improve SPR sensitivity are discussed in other articles and reviews [20–23].The precise mechanism for cells to make such large changes of RI remains unclear. However, detections and/or analyses of cell functions by measuring RI have also been reported by other groups. Chabot *et al.* reported that SPR sensors detected real time adhesion and morphological changes in cells in response to various agents [24]. An SPR sensor based on Fourier Transform infrared FTIR-SPR operating in the near or mid infrared wavelength range was able to monitor changes in cell occupancy and membrane biochemical composition, such as cholesterol [25,26]. Lee *et al.* reported that an SPR sensor combined with olfactory receptor expressing cells provided a new olfactory biosensor system for detection of volatile compounds [27]. Reactions of cancer cells against an anti-cancer drug with SPR sensor have also been reported [28,29]. Maltais *et al.* proposed a label-free assay based on SPR detection of minute morphology changes occurring as a result of apoptosis induction in cells [30]. These studies are summarized in Table 2.

## 4. Application of SPR for Diagnosis of Type I Allergy

The identification of causative antigens that areresponsible for allergic symptoms in patients is crucial in the management of allergic diseases. The histamine release test using peripheral basophils *in vitro* is a safe and sensitive approach. In general, it is more reliable than the detection of antigen-specific IgE in serum. However, basophils of certain individuals do not release histamine, even if they are sensitized with IgE that binds to the antigen, due to dysfunctions in their intracellular signal transduction (non-responder). To overcome such problems, we developed a method to detect SPR signals of peripheral blood basophils. Basophil-enriched leukocytes were purified and fixed on the surface of SPR sensor chip via a monoclonal antibody against a basophil surface antigen.

When basophils sensitized with antigen-specific IgE were fixed on a sensor chip, they immediately caused an increase of RI in response to corresponding antigens, as they did in response to anti-IgE, a positive control stimuli [31,46].

## 5. Diagnosis of Cancer by SPR

**Figure 3.**
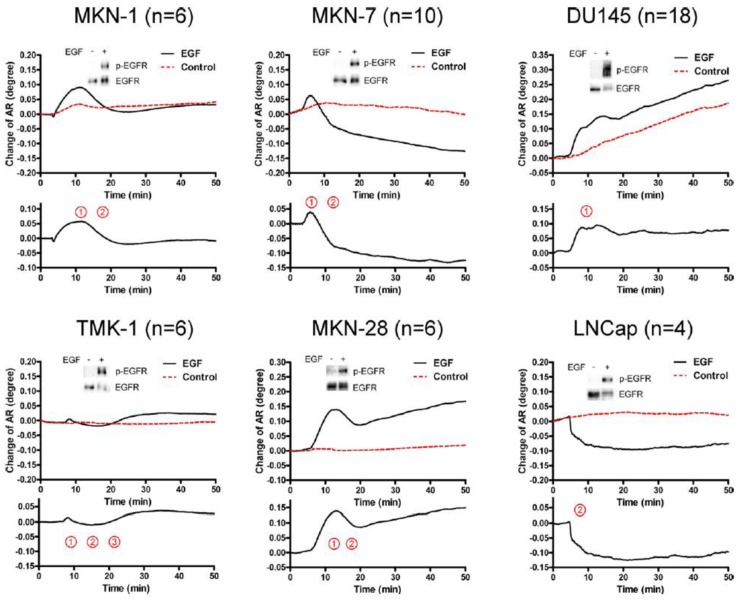
SPR signals (change of angle of resonance (AR)) in six cell lines established from different cancers. TMK-1 cells showed weak, but complete, triphasic changes of AR, whereas the other five cell lines showed unique incomplete patterns of SPR signals. Adapted from [16]

The activation of epidermal growth factor (EGF) receptor (EGFR) on epidermal cells, such as keratinocytes, causes a unique triphasic change of RA, whereas the activation of other receptors, such as the high affinity IgE receptor (FcεRI) on mast cells and basophils, causes a monophasic increase of RA. Chinese hamster ovary (CHO) cells transfected with cDNA for EGFR also showed a triphasic change of RA. However, when CHO cells were transfected with cDNA for EGFR containing a mutation at its kinase domain, they showed a minimal change of RA. Moreover, a phosphatidylinositol 3-kinase inhibitor attenuated the third phase of RA change in CHO cells expressing wild-type EGFR. Furthermore, the pattern of RA change was independent of EGF concentration. These results suggest that EGF induces the SPR signals via the phosphorylation of EGFR, and that an impaired pattern of SPR signal induced by EGF may reveal a disorder in intracellular signal transductions of abnormal cells, such as cancer cells. In fact, we found that five out of six carcinoma cell lines showed mono- or bi-phasic change of RA (Figure 3). These results suggest the potential for the SPR biosensor tobe applied to the real-time detection and/or diagnosis of malignant tumors [16].

## 6. Optic Fiber SPR

**Figure 4.**
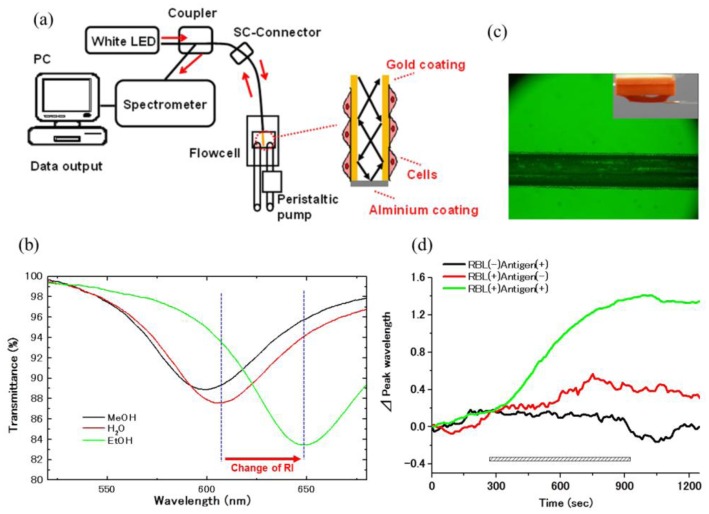
Construction and sensitivity of the optic fiber SPR sensor. (**a**) The optic fiber SPR sensor was composed of a light source (white LED), a plastic cladding multimode optical fiber with quartz core (200/230), fiber connecter (SC), a fiber coupler, a spectrometer, and a personal computer with analysis software. The core of 200 μm diameter with 1cm length of an optical fiber was coated by gold film with 50 nm thickness. (**b**) The absorbance spectra detected in methanol (RI = 1.3265), water (RI = 1.3329) and ethanol (RI = 1.3594). (**c**) RBL-2H3 cells were fixed on the sensor tip surface by means of the droplet method. (**d**) RBL-2H3 cells were cultured on the gold film and caused an increase of RI in response to antigen. Adapted from [32].

The application of an optical fiber sensors-based SPR phenomenon has been reported for the analysis of liquid or gas samples [47,48]. To apply SPR biosensors for the inside of the body, we developed an optic fiber SPR for living cells analysis. The core of 200 μm diameter with 1cm length of an optical fiber was coated by gold film with 50 nm thickness. The light provided by a white LED and attenuated due to an SPR phenomenon in the sensor part was analyzed using a spectrum detector. RIchanges on a gold surface were indicated by shifts of the wavelength of the maximal absorption (Figure 4). Using this sensor, the difference in solvents with various RI and protein bindings to the sensor chip was detected with sufficient sensitivity. Moreover, when RBL-2H3 mast cells were fixed onto the sensor tip surface by means of the droplet method, it detected a sustained increase of RI in response to antigen [32].

## 7. SPR Imaging for Single Cell Analysis

**Figure 5.**
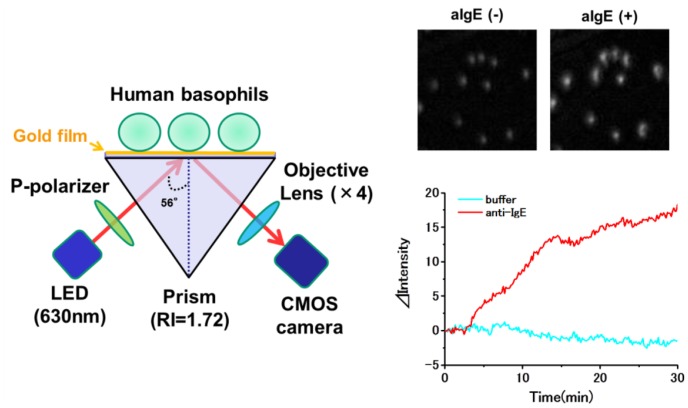
Structure of SPR imaging cell sensor and imaging of human basophils captured with anti-basophilic antibody incubated with or without anti-IgE. Basophils isolated from human peripheral blood were fixed on the surface of sensor chip via an anti-basophilic antibody.

Although SPR sensors possess great potential for revealing nano-scale living cell actions, conventional SPR sensors detect only an average of RI changes in the presence of thousands of cells. Moreover, they can provide only a small number of sensing channels (<10). Therefore, it is difficult to construct an array system for cell activation, and reactions of target cells may be readily overlooked when they are in present in a mixture of different cell types. Furthermore, they cannot reveal the intracellular distribution of RI. We, therefore, have developed a system of SPR imaging (SPRI) that determines a spatial RI distribution of individual cells. The sensor consists of a light source (640 nm LED), CMOS detector, optical prism (RI = 1.72) and a sensor chip with thin gold film (50 nm) matched to the prism via reflected index matching fluid (Figure 5). Using this system, we detected reactions of individual rat mast (RBL-2H3) cells, mouse keratinocytes (PAM212 cells), human epidermal carcinoma (A431) cells, and human basophils (Figure 5) in response to various stimuli, resembling signals obtained by conventional SPR sensors. Moreover, we could distinguish reactions of different types of cells, co-cultured on a sensor chip. It is noteworthy that this system could detect reactions of basophils in response to various antigens in a very small drop of sample (<0.7 μL) [33,34,49]. Horii *et al.* also observed allergic responses of RBL-2H3 cells by using a high magnification 2D-SPR imaging system [35]. Moreover, Shinohara *et al.* applied a 2D-SPR imager for real-time monitoring of translocation of protein kinase C in PC12 cells by measuring RI change [36]. Peterson *et al.* reported a method to monitor interactions of cell-extracellular matrix by SPRI [37,38]. The techniques to detect real-time binding of living cells, such as red blood cells and lymphocytes,to antibodies specific for cell surface antigen coated on SPRI sensor chip were reported by other groups. These studies are summarized in Table 2 [39–43].

## 8. Multiparametric Living Cell Analysis

Since SPR sensors detect whole RI changes in living cells, the information concerningbehavior and function in living cells detected by SPR sensor is limited. Recently, dual biosensing platforms for living cells analysis have been reported. Michaelis *et al.* reported a technique to detect both impedance and RI changes in living cells at the same time using ECIS-SPR sensors [44]. Zhang *et al.* proposed a method for simultaneous measurement of RI distribution and cyclic voltametry, which reflect living cells condition, using electrochemical-surface plasmon resonance imaging (EC-SPRI) [45].These multiparametric analysis techniquescanprovide complementary information regardingliving cells function and behavior.

## 9. Conclusions

SPR and SPRI sensors can detect and visualize living cell reactions and conditions without any labeling. In combination with a device to rapidly isolate cells, such as basophils, lymphocytes, and/or tumor cells which may circulate in human blood, the SPR and SPRI technique should be a useful tool as a high throughput screening system for various clinical diagnoses.

## Figures and Tables

**Table 1.** Biosensors for living cell analysis.

**Technology**	**Target Physical Property**	**Product (Company)**	**[References]**
Impedance sensor	Impedance	xCELLigence (Roche Applied Science) ECIS (Applied Biophysics)	[1,2]
QCM sensor	Mass, Thickness, Viscoelasticity	QCM-D (Q-Sense) Q-sense E4 (Q-sense)	[3,4]
FET sensor	Charge density	-	[5,6]
RWG sensor	Refractive index	Epic system (Corning)	[7,8]
SPR sensor	Refractive index	SPR Cellia (Moritex) Biacore (GE Healthcare)	[9–13]

**Table 2.** Living cell analysis by means of SPR.

**Author (year)**	**Type of SPR**	**Target Cells/Purpose**	**[References]**
Hide (2003)	SPR	RBL-2H3 ^a^/Detection of cells reactions in response to stimuli	[13]
Yanase (2007)	SPR	RBL-2H3 ^a^, PAM212 ^b^, human basophils/Detection of cells reactions in response to stimuli	[14]
Tanaka (2008)	SPR	RBL-2H3 ^a^/Investigation of critical molecules for generation of SPR signal in response to stimuli	[16]
Hiragun (2012)	SPR	Human tumor cells (MKN-1, MKN-7, DU145, TMK-1, MKN-28, LNCap)/Diagnosis of cancer	[17]
Chen (2010)	SPR	GPCR expressing CHO cells (Chinese hamster ovary)	[15]
Chabot (2009)	SPR	HEK-293 ^c^/Detection of adhesion and morphological changes in cells	[24]
Yashunsky (2009)	SPR	MEL 1106 (human melanoma cells)/Monitoring of cell occupancy and membrane biochemical composition	[25]
Ziblat (2006)	SPR	HeLa ^d^/Monitoring of cell occupancy and membrane biochemical composition	[26]
Lee (2009)	SPR	Rat olfactory receptor expressing HEK-293 ^c^/Olfactory biosensor	[27]
Kosaihara (2008) Nishijima (2010)	SPR	MIA PaCa-2, PANC-1,Suit-2 (human pancreatic cancer cell lines)/Detection of cancer cells reaction against an anti-cancer drug	[28,29]
Maltais (2012)	SPR	EA.hy926 (human umbilical vein cells),HeLa ^d^/Detection of apoptosis	[30]
Suzuki (2008)	SPR	Human basophils/Diagnosis of allergy	[31]
Chabot (2012)	LRSPR	HEK-293 ^c^	[18]
Vala (2013)	LRSPR	NRK-52E (rat kidney epithelial cell line)	[19]
Yanase (2010)	Fiber Optic SPR	RBL-2H3 ^a^/Detection of cells reaction in response to stimuli	[32]	
Yanase (2010,2012)	SPRI	RBL-2H3 ^a^, PAM212 ^b^, human basophils/diagnosis of allergy	[33,34]
Horii (2011)	SPRI	RBL-2H3 ^a^/Detection of cells reactions in response to antigen	[35]
Shinohara (2013)	SPRI	PC12 (rat adrenal pheochromocytoma)/detection of cells reactions in response to stimuli	[36]
Peterson (2009, 2010)	SPRI	vSMC (rat aortic vascular smooth muscle cell line)/cell-extracellular matrix interaction	[37,38]
Suraniti (2007)	SPRI	LS102.9 (mouse B-type lymphocytes),13G7 (mouse T-type lymphocytes)/ Detection of cell surface antigen	[39]
Cortès (2011)	SPRI	J774 (murine macrophage cell line), HL-60 (human promyelocytic leukemia cell line) and human PBMC (peritoneal blood mononucleated cell)/Detection of cell surface antigen	[40]
Schasfoort (2013)	SPRI	Human red blood cells/Detection of cell surface antigen	[41]
Stojanović (2014)	SPRI	HS578T,SKBR3, MCF7 (human cancer cell lines)/Detection of cell surface antigen (EpCAM)	[42]
Houngkamhang (2013)	SPRI	Human red blood cells/Detection of cell surface antigen	[43]
Michaelis (2013)	ECIS-SPR	MDCKII (Madin-Darby canine kidney strain II cells)	[44]
Zhang (2013)	EC-SPRI	A549 (Human type II alveolar epithelial cell line)	[45]

a rat basophilic leukemia cell line,

b mouse keratinocyte cell line,

c human embryonic kidney cell line,

d human cervical cancer cells.

## References

[1] Abassi Y.A., Jackson J.A., Zhu J., O'Connell J., Wang X., Xu X. (2004). Label-free, real-time monitoring of IgE-mediated mast cell activation on microelectronic cell sensor arrays. J. Immunol. Methods.

[2] Xi B., Yu N., Wang X., Xu X., Abassi Y.A. (2008). The application of cell-based label-free technology in drug discovery. Biotechnol. J..

[3] Marx K.A., Zhou T., Montrone A., Schulze H., Braunhut S.J. (2001). A quartz crystal microbalance cell biosensor: Detection of microtubule alterations in living cells at nM nocodazole concentrations. Biosens. Bioelectron..

[4] Saitakis M., Gizeli E. (2012). Acoustic sensors as a biophysical tool for probing cell attachment and cell/surface interactions. Cell Mol. Life Sci..

[5] Sakata T., Miyahara Y. (2008). Noninvasive monitoring of transporter-substrate interaction at cell membrane. Anal. Chem..

[6] Sakata T., Sugimoto H. (2011). Continuous monitoring of electrical activity of pancreatic β-cells using cell-based field effect transistor. Jpn. J. Appl. Phys..

[7] Fang Y., Ferrie A.M., Fontaine N.H., Yuen P.K. (2005). Characteristics of dynamic mass redistribution of epidermal growth factor receptor signaling in living cells measured with label-free optical biosensors. Anal. Chem..

[8] Fang Y., Ferrie A.M., Tran E. (2009). Resonant waveguide grating biosensor for whole-cell GPCR assays. Methods Mol. Biol..

[9] Homola J. (2003). Present and future of surface plasmon resonance biosensors. Anal. Bioanal. Chem..

[10] Szabo A., Stolz L., Granzow R. (1995). Surface plasmon resonance and its use in biomolecular interaction analysis (BIA). Curr. Opin. Struct. Biol..

[11] Ritzefeld M., Sewald N. (2012). Real-time analysis of specific protein-DNA interactions with surface plasmon resonance. J. Amino. Acids..

[12] Abdulhalim I., Zourob M., Lakhtakia A. (2008). Surface plasmon resonance for biosensing. Electromagnetics.

[13] Hide M., Tsutsui T., Sato H., Nishimura T., Morimoto K., Yamamoto S., Yoshizato K. (2002). Real-time analysis of ligand-induced cell surface and intracellular reactions of living mast cells using a surface plasmon resonance-based biosensor. Anal. Biochem..

[14] Yanase Y., Suzuki H., Tsutsui T., Hiragun T., Kameyoshi Y., Hide M. (2007). The SPR signal in living cells reflects changes other than the area of adhesion and the formation of cell constructions. Biosens. Bioelectron..

[15] Chen K., Obinata H., Izumi T. (2010). Detection of G protein-coupled receptor-mediated cellular response involved in cytoskeletal rearrangement using surface plasmon resonance. Biosens. Bioelectron..

[16] Tanaka M., Hiragun T., Tsutsui T., Yanase Y., Suzuki H., Hide M. (2008). Surface plasmon resonance-biosensor detects the downstream events of active PKCβ in antigen-stimulated mast cells. Biosens. Bioelectron..

[17] Hiragun T., Yanase Y., Kose K., Kawaguchi T., Uchida K., Tanaka S., Hide M. (2012). Surface plasmon resonance-biosensor detects the diversity of responses against epidermal growth factor in various carcinoma cell lines. Biosens. Bioelectron..

[18] Chabot V., Miron Y., Grandbois M., Charette P.G. (2012). Long range surface plasmon resonance for increased sensitivity in living cell biosensing through greater probing depth. Sens. Actuators B.

[19] Vala M., Robelek R., Bocková M., Wegener J., Homola J. (2013). Real-time label-free monitoring of the cellular response to osmotic stress using conventional and long-range surface plasmons. Biosens. Bioelectron..

[20] Lahav A., Auslender M., Abdulhalim I. (2008). Sensitivity enhancement of guided wave surface plasmon resonance sensors. Opt. Lett..

[21] Krasnykov O., Karabchevsky A., Shalabney A., Auslender M., Abdulhalim I. (2011). Sensor with increased sensitivity based on enhance doptical transmission in the infrared. Opt. Commun..

[22] Shalabney A., Abdulhalim I. (2011). Sensitivity enhancement methods for surface plasmon sensors. Lasers Photon. Rev..

[23] Shalabney A., Abdulhalim I. (2012). Figure of merit enhancement of surface plasmon resonance sensors in the spectral interrogation. Opt. Lett..

[24] Chabot V., Cuerrier C.M., Escher E., Aimez V., Grandbois M., Charette P.G. (2009). Biosensing based on surface plasmon resonance and living cells. Biosens. Bioelectron..

[25] Yashunsky V., Shimron S., Lirtsman V., Weiss A.M., Melamed-Book N., Golosovsky M., Davidov D., Aroeti B. (2009). Real-time monitoring of transferrin-induced endocytic vesicle formation by mid-infrared surface plasmon resonance. Biophys. J..

[26] Ziblat R., Lirtsman V., Davidov D., Aroeti B. (2006). Infrared surface plasmon resonance: A novel tool for real time sensing of variations in living cells. Biophys. J..

[27] Lee S.H., Ko H.J., Park T.H. (2009). Real-time monitoring of odorant-induced cellular reactions using surface plasmon resonance. Biosens. Bioelectron..

[28] Kosaihira A., Ona T. (2008). Rapid and quantitative method for evaluating the personal therapeutic potential of cancer drugs. Anal. Bioanal. Chem..

[29] Nishijima H., Kosaihira A., Shibata J., Ona T. (2010). Development of signaling echo method for cell-based quantitative efficacy evaluation of anti-cancer drugs in apoptosis without drug presence using high-precision surface plasmon resonance sensing. Anal. Sci..

[30] Maltais J.S., Denault J.B., Gendron L., Grandbois M. (2012). Label-free monitoring of apoptosis by surface plasmon resonance detection of morphological changes. Apoptosis.

[31] Suzuki H., Yanase Y., Tsutsui T., Ishii K., Hiragun T., Hide M. (2008). Applying surface plasmon resonance to monitor the IgE-mediated activation of human basophils. Allergol. Int..

[32] Yanase Y., Araki A., Suzuki H., Tsutsui T., Kimura T., Okamoto K., Nakatani T., Hiragun T., Hide M. (2010). Development of an optical fiber SPR sensor for living cell activation. Biosens. Bioelectron..

[33] Yanase Y., Hiragun T., Kaneko S., Gould H., Greaves M., Hide M. (2010). Detection of refractive index changes in individual cells by means of surface plasmon resonance imaging. Biosens. Bioelectron..

[34] Yanase Y., Hiragun T., Yanase T., Kawaguchi T., Ishii K., Hide M. (2012). Evaluation of peripheral blood basophil activation by means of surface plasmon resonance imaging. Biosens. Bioelectron..

[35] Horii M., Shinohara H., Iribe Y., Suzuki M. (2011). Living cell-based allergen sensing using a high resolution two-dimensional surface plasmon resonance imager. Analyst.

[36] Shinohara H., Sakai Y., Mir T.A. (2013). Real-time monitoring of intracellular signal transduction in PC12 cells by two-dimensional surface plasmon resonance imager. Anal. Biochem..

[37] Peterson A.W., Halter M., Tona A., Bhadriraju K., Plant A.L. (2009). Surface plasmon resonance imaging of cells and surface-associated fibronectin. BMC Cell Biol..

[38] Peterson A.W., Halter M., Tona A., Bhadriraju K., Plant A.L. (2010). Using surface plasmon resonance imaging to probe dynamic interactions between cells and extracellular matrix. Cytometry A.

[39] Suraniti E., Sollier E., Calemczuk R., Livache T., Marche P.N., Villiers M.B., Roupioz Y. (2007). Real-time detection of lymphocytes binding on an antibody chip using SPR imaging. Lab Chip.

[40] Cortès S., Villiers C.L., Colpo P., Couderc R., Brakha C., Rossi F., Marche P.N., Villiers M.B. (2011). Biosensor for direct cell detection, quantification and analysis. Biosens. Bioelectron..

[41] Schasfoort R.B., Bentlage A.E., Stojanovic I., van der Kooi A., van der Schoot E., Terstappen L.W., Vidarsson G. (2013). Label-free cell profiling. Anal. Biochem..

[42] Stojanović I., Schasfoort R.B., Terstappen L.W. (2014). Analysis of cell surface antigens by surface plasmon resonance imaging. Biosens. Bioelectron..

[43] Houngkamhang N., Vongsakulyanon A., Peungthum P., Sudprasert K., Kitpoka P., Kunakorn M., Sutapun B., Amarit R., Somboonkaew A., Srikhirin T. (2013). ABO blood-typing using an antibody array technique based on surface plasmon resonance imaging. Sensors.

[44] Michaelis S., Wegener J., Robelek R. (2013). Label-free monitoring of cell-based assays: Combining impedance analysis with SPR for multiparametric cell profiling. Biosens. Bioelectron..

[45] Zhang L.L., Chen X., Wei H.T., Li H., Sun J.H., Cai H.Y., Chen J.L., Cui D.F. (2013). An electrochemical surface plasmon resonance imaging system targeting cell analysis. Rev. SciInstrum..

[46] Yanase Y., Suzuki H., Tsutsui T., Uechi I., Hiragun T., Mihara S., Hide M. (2007). Living cell positioning on the surface of gold film for SPR analysis. Biosens. Bioelectron..

[47] Nelson R.W., Krone J.R., Jansson O. (1997). Surface Plasmon resonance biomolecular interaction analysis mass spectrometry. 2. Fiber optic-based analysis. Anal. Chem..

[48] Mitsushio M., Higashi S., Higo M. (2004). Construction and evaluation of a gold-deposited optical fiber sensor system for measurements of refractive indices of alcohols. Sens. Actuators A.

[49] Yanase Y., Hiragun T., Yanase T., Kawaguchi T., Ishii K., Hide M. (2013). Application of SPR imaging sensor for detection of individual living cell reactions and clinical diagnosis of type I allergy. Allergol. Int..

